# Comparison of the direct and indirect reduction techniques during the surgical management of posterior malleolar fractures

**DOI:** 10.1186/s12891-017-1475-7

**Published:** 2017-03-14

**Authors:** Hong-fei Shi, Jin Xiong, Yi-xin Chen, Jun-fei Wang, Xu-Sheng Qiu, Jie Huang, Xue-yang Gui, Si-yuan Wen, Yin-he Wang

**Affiliations:** 1 Department of Orthopaedics, Nanjing Drum Tower Hospital, The Affiliated Hospital of Nanjing University Medical School, No. 321 Zhongshan Road, Nanjing, China; 2 Nanjing Medical University, Nanjing, China; 3 Nanjing University Medical School, Nanjing, China

**Keywords:** Posterior malleolar fracture, Posterolateral approach, Buttress plate, Ligamentotaxis

## Abstract

**Background:**

The optimal method for the reduction and fixation of posterior malleolar fracture (PMF) remains inconclusive. Currently, both of the indirect and direct reduction techniques are widely used. We aimed to compare the reduction quality and clinical outcome of posterior malleolar fracture managed with the direct reduction technique through posterolateral approach or the indirect reduction technique using ligamentotaxis.

**Methods:**

Patients with a PMF involving over 25% of the articular surface were recruited and assigned to the direct reduction (DR) group or the indirect reduction (IR) group. Following reduction and fixation of the fracture, the quality of fracture reduction was evaluated in post-operative CT images. Clinical and radiological follow-ups were performed at 6 weeks, 3 months, 6 months, 12 months, and then at 6 month-intervals postoperatively. Functional outcome (AOFAS score), ankle range of motion, and Visual Analog Scale (VAS) were evaluated at the last follow-up. Statistical differences were compared between the DR and IR groups considering the patient demographics, quality of fracture reduction, AOFAS score, and VAS.

**Results:**

Totally 116 patients were included, wherein 64 cases were assigned to the DR group and 52 cases were assigned to the IR group. The quality of fracture reduction was significant higher in the DR group (*P* = 0.038). In the patients who completed a minimum of 12 months’ follow-up, a median AOFAS score of 87 was recorded in the DR group, which was significantly higher than that recorded in the IR group (a median score of 80). The ankle range of motion was slightly better in the DR group, with the mean dorsiflexion restriction recorded to be 5.2° and 6.1° in the DR and IR group respectively (*P* = 0.331). Similar VAS score was observed in the two groups (*P* = 0.419).

**Conclusions:**

The direct reduction technique through a posterolateral approach provide better quality of fracture reduction and functional outcome in the management of PMF over 25% of articular surface, as compared with the indirect reduction technique using ligamentotaxis.

**Trial registration:**

NCT02801474 (retrospectively registered, June 2016, ClinicalTrails.gov).

## Background

The incidence of the posterior malleolar fracture (PMF) was reported to be 7 to 44% of all ankle fractures [1]. Despite of the evidence provided by anatomical and biomechanical studies that the posterior malleolus serves as an important contributor to the stability of ankle mortise and syndesmosis, the indication of surgical reduction and fixation of PMF remains controversial [2–4]. Fragment size is frequently used as a reference for surgical intervention, and the posterior malleolar fracture involves more than 25% of articular surface is generally recommended to be reduced and fixed to prevent instability and reduce the risk of post-traumatic degenerative changes [1].

The optimal method for the reduction and fixation of PMF remains inconclusive. Often, the posterior fragment, also known as Volkmann’s fragment, reduces simultaneously via ligamentotaxis of the posteroinferior tibiofibular ligament (PITL) following an anatomical reduction of the lateral malleolus [5]. Following this indirect reduction procedure, percutaneous screw fixation can be achieved through a stab incision. Alternatively, the posterior malleolus fragment can be direct visualized and reduced through a posterolateral approach with the patient in a prone position [6]. Buttress plate or lag screws can then be placed to stabilize the fragment.

Currently, both of the indirect and direct reduction techniques are widely used among orthopaedic surgeons during the operative treatment of PMF, while conflicting data has been published regarding the subsequent clinical outcomes [7–10]. Few studies could be found comparing the quality of fracture reduction, as well as the functional and clinical outcomes between the indirect and direct reduction techniques. In this study, we aimed to compare the reduction quality and clinical outcome of PMF managed with the direct reduction technique through a posterolateral approach or the indirect reduction technique using ligamentotaxis.

## Methods

This study was approved by the ethics committee of the authors’ institution (Ref. No. 115690). Between January 2012 and December 2014, patients with a posterior malleolus fracture were recruited as candidates for this prospective study. Following screening by two of the senior surgeons (HFS and XSQ), the patients were finally included meeting the inclusion criteria of (1) age between18 to 70 years, (2) unstable and displaced (more than 2 mm) posterior malleolar fractures requiring surgical management, (3) posterior malleolar fragment involving over 25% of the articular surface measured in preoperative lateral view radiographs and sagittal reconstruction of CT images. Patients with open fractures, pathological fractures, delayed fractures, or additional ipsilateral lower extremity fractures were excluded. Informed-consent documents were obtained from all the eligible patients that stated the aim and protocol of this study. Preoperative anteroposterior (AP), lateral, and mortise view radiographs as well as computed tomography (CT) scans were routinely obtained to evaluate the characteristic of the fracture according to the Lauge-Hansen classification and AO/OTA classification. The injury mechanism was recorded as low-energy injury (a small-level fall or a sprain) or high-energy injury (a motor-vehicle accident or a fall from a height > 1 meter). Patients were then assigned to the direct reduction (DR) group or the indirect reduction (IR) group by monthly turns.

**Fig. 1:**
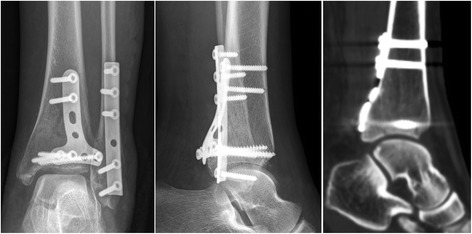
For the patient presented in Fig. 4, a distal radius plate was applied spanning the fracture in a buttress mode to fix the PMF. The postoperative sagittal reconstruction of CT images confirmed an excellent (anatomical) reduction of the posterior fragment

All the patients were operated on by two of the senior surgeons (JX and YXC). During operation, patients received general or spinal anesthesia, with a thigh tourniquet applied to prevent bleeding. In the DR group, the posterior and lateral malleoli were accessed via a posterolateral approach with the patients in prone position [11]. Care was taken to protect the sural nerve and lesser saphenous vein during superficial dissection [12]. With the peroneal tendons retracted medially, the fibular fracture was exposed and reduced anatomically in the first place. Posteriorly placed one-third tubular plate, reconstruction plate, or laterally placed posterolateral distal fibula plate (Synthes, Switzerland) was used to stabilize the fibular fracture. The posterior malleolus was then exposed between the fiexor halluces longus and peroneus longus interval. The PITL was carefully preserved. Articular impaction was inspected and reduced if any. The posterior malleolar fragment was then reduced with reference to the typical metaphyseal-diaphyseal spike of the posterior malleolus. If comminution occurred, the fracture reduction was performed in a lateral-to-medial manner. One-third tubular plate, reconstruction plate, or distal radius plate (Synthes, Switzerland) were applied spanning the fracture in a buttress mode (Fig. 1). One or multiple 4.0 mm cannulated screws could also be used if applicable. A separate medial approach was then performed to address the medial osseous and/or ligamentous injuries. Anatomical reduction and fixation of the medial malleolus was achieved under direct visualization and verified using intraoperative C-arm.

In the IR group, the lateral and/or medial malleolar fractures received open reduction and internal fixation (ORIF) using standard AO (Arbeitsgemeinschaft für Osteosynthesefragen) osteosynthesis technique with the patients in supine position. Anatomical reduction of the lateral and/or medial malleolus was confirmed under fluoroscopy. The posterior malleolus was then reduced through ligamentotaxis with the ankle in dorsiflexion. If necessary, percutaneously applied pointed reduction forceps, bone hook, K-wire, or periosteal elevator was used to reduce the posterior malleolar fragment anatomically [13, 14]. Intraoperative lateral view radiographs were taken using C-arm to verify the reduction. One or two 4.0 mm cannulated screws were then used to fix the posterior malleolar in anterior-to-posterior direction.

In both groups, intraoperative external rotation stress test and Cotton test were performed to evaluate the stability of syndesmosis following reduction and fixation of the fractures. Syndesmotic screws were placed when positive results were observed in the external rotation stress test (increased medial clear space at the ankle mortise) or in the Cotton test (>2 mm of lateral migration of the lateral malleolus) [15, 16].

Postoperatively, all the fractures were stabilized with a short leg splint for 2 weeks, followed by 4 weeks of non-weight-bearing rehabilitation focusing on the range of motion. Regular clinical and radiological assessments were performed at 6 weeks, 3 months, 6 months, 12 months, and then at 6 month-intervals according to our routine follow-up regime. Progressive weight bearing and strengthening was initiated since 6 weeks postoperatively, with reference to the healing status checked in follow-up radiographs. The syndesmotic screws were removed, if any, at 3 months postoperatively.

Postoperative radiographs and CT scan were obtained routinely to evaluate the quality of fracture reduction. The maximum residual displacement of the posterior malleolus, the gap in the joint surface, and/or the articular step-off were measured in sagittal cuts of the CT scan. The quality of fracture reduction was graded as excellent (less than 1 mm), good (1 to 2 mm), or poor (more than 2 mm) as described in literature [17].

Functional outcome was evaluated according to the American Orthopaedic Foot and Ankle Society ankle-hindfoot score (AOFAS) at the last follow-up [18]. The ankle range of motion (ROM) was measured, and the difference of the dorsiflexion between the injured and uninjured side was calculated and presented as dorsiflexion restriction [19, 20]. Visual Analog Scale (VAS) was used to evaluate local pain.

All the evaluations were performed by two of the senior surgeons (JFW and YHW) independently. The interobserver quantitative data were averaged for further statistical analysis. IBM SPSS version 19.0 software (SPSS Inc., Chicago, IL) was used with the statistical significance set at a *P* value of less than 0.05. The assumption of normal distribution of the parametric data was testified using Q-Q plots. Demographics of the two groups were compared using independent *t* test or Chi-Square test. The quality of fracture reduction were compared using Chi-Square test. The AOFAS score and VAS were analyzed using Mann-Whitney *U* test to compare the difference between groups.

## Results

The demographics of the two groups were presented in Table 1. Totally 116 patients (116 fractures) were included in this investigation, among which 64 cases were assigned to the DR group and 52 cases were assigned to the IR group. No significant difference was detected between the groups considering the age (*P* = 0.751), gender (*P* = 0.097), Lauge-Hansen classification (*P* = 0.647), AO/OTA classification (*P* = 0.344), injury mechanism (*P* = 0.642), and the time from trauma to surgery (*P* = 0.733).

**Table 1:** Patient demographics and results

	Direct reduction group	Indirect reduction group	*P* value
No. of patients	64	52	
Gender			
Male	28	31	0.097
Female	36	21	
Lauge-Hansen classification			
Pronation-external rotation	12	12	0.647
Supination-external rotation	52	40	
AO/OTA classification			
44B3	47	35	0.344
44C1	4	3	
44C2	11	13	
44C3	2	1	
Injury mechanism			
Low-energy	50	43	0.642
High-energy	14	9	
Age (*Yr.*)	49.0 ± 12.4	48.1 ± 15.2	0.751
Time before surgery (days)	4.3 ± 2.0	4.5 ± 2.8	0.733
Quality of reduction			
Excellent	34 (53.1%)	16 (30.8%)	0.038*
Good	25 (39.1%)	27 (51.9%)	
Poor	5 (7.8%)	9 (17.3%)	
Follow-up duration (months)	19.9 ± 5.2	20.0 ± 5.8	0.882
AOFAS	87 (58 to 95)	80 (59 to 95)	0.034*
Dorsiflexion restriction (°)	5.2 ± 4.5	6.1 ± 4.3	0.331
VAS	2 (0 to 7)	2 (0 to 7)	0.419

*P < 0.05

As measured in the postoperative radiographs and CT scan, 34 (53.1%) and 16 (30.8%) patients achieved excellent fracture reduction in the DR and IR group respectively (Table 1). The percentage of excellent fracture reduction was 22.3% higher in the DR group than in the IR group. A good fracture reduction was observed in 25 (39.1%) patients in the DR group and in 27 (51.9%) patients in the IR group. The percentage of excellent or good reduction was 9.5% higher in the DR group than in the IR group. More than 2 mm residual fracture displacement and/or articular step-off were found in 5 (7.8%) patients in the DR group and in 9 (17.3%) patients in the IR group. The quality of fracture reduction was significant higher in the DR group compared with that in the IR group (*P* = 0.038).

**Fig. 2:**
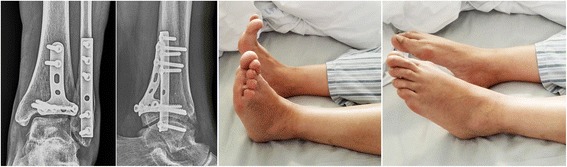
For the patient presented in Fig. 4, the AOFAS score was 94 at the last follow-up (24 months postoperatively). Satisfied ankle range of motion were achieved

**Fig. 3:**
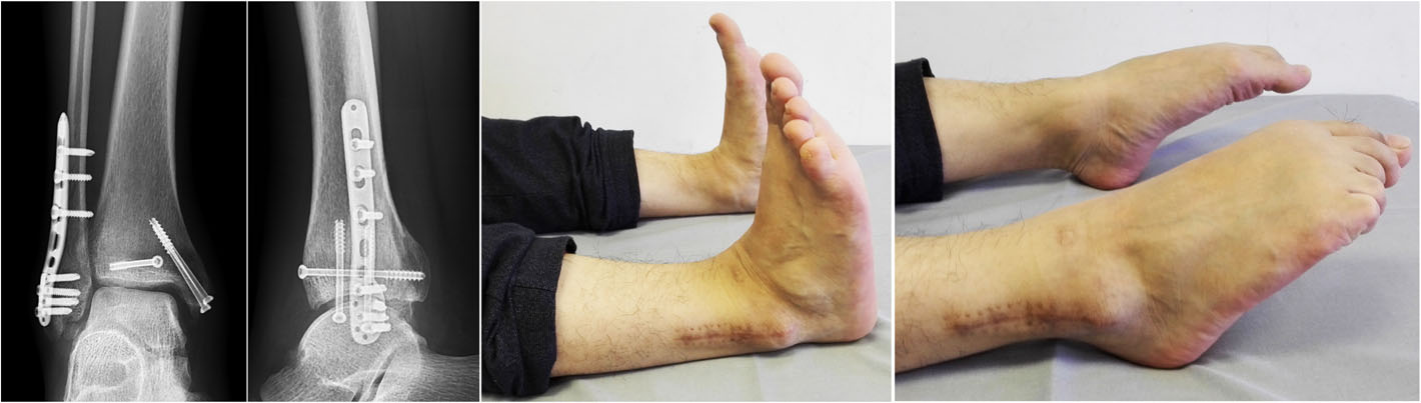
For the patient presented in Fig. 5, the AOFAS score was 88 at the last follow-up (12 months postoperatively). Satisfied ankle range of motion were achieved

Totally 89 patients (48 in the DR group and 41 in the IR group) completed a minimum of 12 months’ follow-up with the clinical outcome evaluated in the outpatient clinic (Figs. 2 and 3). The mean follow-up duration was 19.9 (12 to 32) months and 20.0 (12 to 36) months in the DR and IR group respectively (*P* = 0.882). The other 27 patients were lost to follow-up due to different kind of reasons. At the last follow-up, a median AOFAS score of 87 was recorded in the DR group, which was significantly higher than that recorded in the IR group (a median score of 80). The ROM of the ankle was slightly better in the DR group, with the mean dorsiflexion restriction recorded to be 5.2° and 6.1° in the DR and IR group respectively (*P* = 0.331). Besides, similar VAS score was observed in the two groups (*P* = 0.419).

No major intraoperative complications was observed. Two patient in the DR group developed superficial infection postoperatively. Both of them were managed successfully with local dressing changes and oral antibiotics. Calf muscle vein thrombosis was diagnosed in one patient in the DR group and two patients in the IR group during follow-up. Therapeutic dosages of low-molecular-weight heparins (LMWH) was prescribed till thrombolysis confirmed using color duplex Doppler ultrasound. Fracture nonunion, implant loosening or failure was not observed in each of the groups.

## Discussion

Surgical management of ankle fractures aims to achieve articular congruity, ankle alignment and stability with maximal functional recovery. Unlike the studies on the management of lateral and medial malleolar fractures, the surgical indication and optimal technique for the management of PMF remains inconclusive. In the study, we compared the radiological and functional outcome of PMF managed with the direct or indirect reduction technique. Higher quality of fracture reduction and better functional outcome were achieved when the PMF was treated with direct reduction technique through a posterolateral approach, while similar ankle range of motion and VAS score were observed when compared with the patients managed with the indirect reduction technique using ligamentotaxis.

**Fig. 4:**
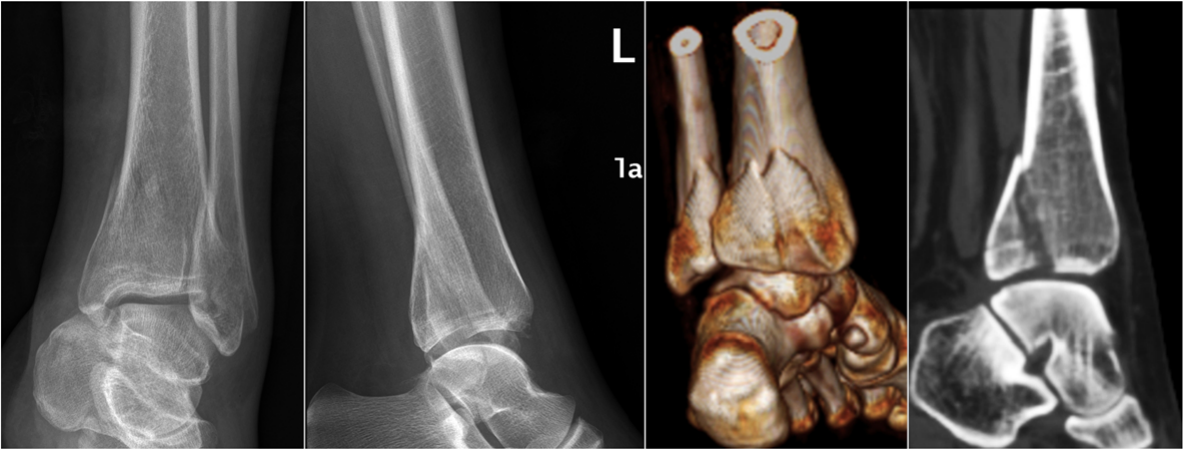
The 56-year-old female patient treated using direct reduction technique. Preoperative AP and lateral radiograph showed a pilon-type PMF. Preoperative 3D and sagittal reconstruction of CT images provided more precise determination of the morphology and size of the posterior fragments

**Fig. 5:**
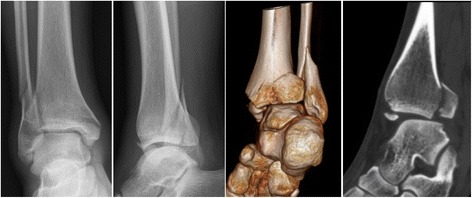
The 55-year-old male patient treated using indirect reduction technique. Preoperative radiographs and CT images showed a displaced PMF

Currently, the recommended indication for posterior malleolar fixation is when the fracture affecting over 25% of the articular surface, displacement over 2 mm, ankle instability, and persistent subluxation of the talus [4]. In our study, we included the patients with a posterior malleolar fragment involving over 25% of the articular surface measured in preoperative lateral view radiographs or sagittal reconstruction of CT images (Figs. 4 and 5). The debate continues whether the fragment less than 25% should be anatomically reduced and fixed. Some authors reported negative relationships between the fixation of small posterior malleolar fragment and the functional outcome, while others believed that the fixation of small posterior malleolar fragment would contribute to the stability of syndesmosis [2, 4, 21, 22]. Recent anatomical and clinical studies provide evidence that the morphology of the fracture fragment might be more important than the fragment size [1, 23]. A thorough study of the morphology of the fracture fragment in reconstructed CT images might provide information about the injury mechanisms, and hence guided different way of reduction and fixation [9, 24].

**Fig. 6:**
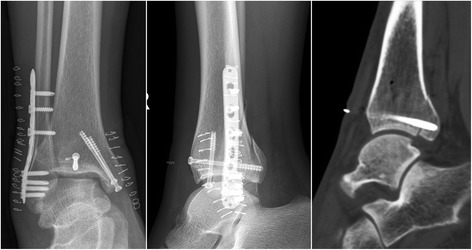
For the patient presented in Fig. 5, the PMF was reduced using ligamentotaxis following ORIF of lateral and medial malleoli. Percutaneous screw fixation of PMF was achieved through a stab incision. The postoperative sagittal reconstruction of CT images provided more precise determination of residual articular step-off of the posterior malleolus which could be easily ignored in radiographs

One important finding of our study was that the direct reduction technique through a posterolateral approach led to significant higher quality of fracture reduction, as evaluated in postoperative CT images, than the indirect reduction technique using ligamentotaxis. Since plain radiography was considered inaccurate with poor reliability in the assessment of PMF, postoperative CT scan was routinely performed in our institution to evaluate the quality of fracture reduction [25, 26]. The rate of excellent (or anatomical) reduction were observed to be 53.1% (34/64) and 30.8% (16/52) in the DR and IR group respectively. In Huber’s study, the rate of anatomical reduction of PMF was reported to be 27% via indirect reduction, while 83% of the patients achieved anatomical reduction using direct posterior approach [6]. A possible reason for the remarkable lower rate of anatomical reduction observed in our study compared with that in Huber’s study was that CT scan might provide more precise determination of residual fracture gap and articular step-off of posterior malleolus [9] (Fig. 6).

Several factors might contribute to the better quality of fracture reduction achieved via the direct reduction technique. First, it’s more convenient to use the typical metaphyseal-diaphyseal spike of the posterior malleolus as reference to reduce the posterior fragment when exposed through the posterolateral approach. Second, fixation of small posterior malleolar fragment to larger distal tibia might be biomechanically stronger than anterior-to-posterior screw fixation, which would help to achieve better interfragmentary compression and reduce gaps between the posterior fragment and distal tibia [6]. Gravity would also help to reduce the talus and PMF in prone position. In case of the occurrence of articular impaction, the posterior lateral approach might provide adequate visualization of the posterior fragment compared with the indirect reduction technique. The impacted articular fragment could then be meticulously accessed and reduced through fracture line or via cortical window.

Despite of the advantages described above, the direct reduction technique did not prevail over the indirect reduction technique in clinical practice [7]. It was reported that 83% of posterior malleolar fractures were fixed using anterior-to-posterior screws with indirect reduction technique. [8] Some authors believed that indirect reduction and percutaneous screw fixation were less traumatic, while posterolateral approach might increase the risk of posterior scarring, tendon impingement, and sural nerve injury [27, 28]. Provided that PITL was intact, the posterolateral fragment could be easily reduced using ligamentotaxis following anatomical reduction of the fibula [1]. However, when the PMF was severely comminuted, impacted, or accompanied by medial extension (described as Haraguchi type-II fracture), direct reduction technique was preferred [29] (Fig. 1).

Functional outcome of ankle fractures was believed to be associated with fracture morphology, articular involvement, fracture fragment size, severity of comminution, and residual step-off or gaps. In our study, provided a better quality of fracture reduction, the DR group presented significantly higher AOFAS score than the IR group. The ankle range of motion, on the other hand, was only slightly better in the DR group, as reflected by the recorded dorsiflexion restriction. In literature, very different results were reported considering the functional outcome of PMF following surgical reduction and fixation. Choi reported a mean AOFAS score of 90.6 following ORIF of PMF through a modified posterolateral approach [28]. Erdem achieved a mean AOFAS score of 94.1 of PMF following posterior-to-anterior lag screw fixation or buttress plate fixation through posterolateral approach [30]. Compared with our study, the patients with a PMF smaller than 25% of articular surface were included in these studies, which might contribute to the higher AOFAS scores reported in these studies. Besides, the pilon-type PMFs, characterized by posteromedial fracture extension to the medial malleolus with varying articular impaction, were exclude from Erdem’s study [29]. The pilon-type PMFs were generally believed to lead to inferior functional outcomes than regular ankle fractures [31–33]. Evers and Drijfhout van Hooff reported mean AOFAS scores of 70.9 and 81 in their studies respectively, wherein the PMFs greater than 25% of the joint surface were fixed [9, 20]. These results were similar with our study, while detailed techniques of fracture reduction and fixation, unfortunately, were not described in these two studies.

In this study, as one of the limitations, the radiographic osteoarthritis status was not graded or compared between groups due to limited time of follow-up [10]. Theoretically, an anatomically reduced and fixed posterior malleolus would provide important buttress to contain the talus, stabilize syndesmosis, and reduce the risk of post-traumatic osteoarthritis [20]. Future study with long-term follow-up results would provide further information considering the influence of different rate of anatomical reduction on the grade of post-traumatic arthrosis between the DR and the IR groups.

## Conclusions

In conclusion, the direct reduction technique through a posterolateral approach provide better quality of fracture reduction and functional outcome in the management of PMF over 25% of articular surface, as compared with the indirect reduction technique using ligamentotaxis.

## Abbreviations

AOFAS: American Orthopaedic Foot and Ankle Society
AP: Anteroposterior
CT: Computed tomography
DR: Direct reduction
IR: Indirect reduction
LMWH: Low-molecular-weight heparins
ORIF: Open reduction and internal fixation
PITL: Posteroinferior tibiofibular ligament
PMF: Posterior malleolar fracture
ROM: Range of motion
VAS: Visual Analog Scale
